# Rotavirus and adenovirus frequency among patients with acute gastroenteritis and their relationship to clinical parameters: a retrospective study in Turkey

**DOI:** 10.1186/1447-056X-8-8

**Published:** 2009-11-29

**Authors:** Hülya Akan, Güldal İzbırak, Yesim Gürol, Sezgin Sarıkaya, Tehlile S Gündüz, Gülden Yılmaz, Osman Hayran, Ayca Vitrinel

**Affiliations:** 1 Department of Family Medicine, Yeditepe University Faculty of Medicine, İnönü Mahallesi, Kayışdağı Cad, 26 Ağustos Yerleşimi, 34755 Kadıköy, İstanbul, Turkey; 2 Department of Microbiology and Clinical Microbiology, Yeditepe University Faculty of Medicine, İnönü Mahallesi, Kayışdağı Cad, 26 Ağustos Yerleşimi, 34755 Kadıköy, İstanbul, Turkey; 3 Department of Emergency Medicine, Yeditepe University Faculty of Medicine, İnönü Mahallesi, Kayışdağı Cad, 26 Ağustos Yerleşimi, 34755 Kadıköy, İstanbul, Turkey; 4 Faculty of Health Sciences, Yeditepe University Faculty of Medicine, İnönü Mahallesi, Kayışdağı Cad, 26 Ağustos Yerleşimi, 34755 Kadıköy, İstanbul, Turkey; 5 Department of Pediatric Child Health and Diseases, Yeditepe University Faculty of Medicine, İnönü Mahallesi, Kayışdağı Cad, 26 Ağustos Yerleşimi, 34755 Kadıköy, İstanbul, Turkey

## Abstract

**Background:**

Diarrhea is the third leading cause of death related to infectious diseases all over the world. The diseases related to viral gastroenteritis are gradually increasing, particularly in the developed countries. The purpose of our study was to determine the frequency and to investigate the clinical manifestations of acute rotavirus and adenovirus gatroenteritis and to assess the diagnostic value of the related clinical findings.

**Methods:**

In 2007-2008 patients with diarrhea and/or vomiting attended to Yeditepe University Hospital and related clinics, Istanbul, were studied. The rotavirus and/or adenovirus antigen in stool of these patients were investigated. Data regarding clinical findings were collected from the electronic records, retrospectively. Age, gender, symptoms, fever, antibiotic use, vomiting, number of vomiting and diarrhaeae, dehydration, abdominal pain, the other pathological physical examination findings were analyzed by the physicians in the study group. To investigate the rotavirus and adenovirus antigen CerTest Rota-Adeno Blister Test (CerTest, Biotec, Spain), a qualitative immunochromotographic assay was used. Statistical analysis wasperformed with SPSS v. 11,5 statistical software. X^2 ^test was used for bivariate and logistic regression analysis was used for multivariate analysis.

**Results:**

Rotavirus positivity was 18,7% (n = 126). Concomitantly, in 596 cases adenovirus antigen test were also performed. Adenovirus positivity was 8,9% (n = 53) and rota-adenovirus co-infection was 4,4% (n = 26). Most of rotavirus positive cases were seen in December, January, February and March (p < 0.001). In clinical parameters, there was a significant difference between rotavirus positive cases and negative cases regarding to vomiting, dehydration and vomiting and diarrhea coexistence (respectively p = 0.010, p < 0.00, p = 0.007).

**Conclusion:**

Rotavirus can be seen in all age groups, but more frequently in childhood. Although there is no clinical gold standard to distinguish the rotavirus cases from the other gastroenteritis agents, the findings of dehydration and vomiting-diarrhea coexistence, considering months of referral may lead clinician to perform rapid antigen tests and affect approach to the treatment. Prospective studies with representative samples are needed to determine the rotavirus and adenovirus incidence and to develop safe and reliable protective policies in our country.

## Background

Diarrhea is the third leading cause of death related to infectious diseases all over the world; the rate of death due to diarrheal diseases is estimated as two millions a year (1.7 - 2.5 millions) [1].

All of the bacteria, parasites, and viral pathogens are considered among the causes of infectious gastroenteritis [2]. The wide diversity of bacterial and viral infections that may cause diarrhea complicates accurate surveillance and diagnosis, especially in developing countries [3]. Gastroenteritis caused by viral agents are gradually increasing particularly in the developed countries. Although the improvements in sanitation have significantly decreased the gastroenteritis cases caused by bacteria and parasites, it has little effect on viral gastroenteritis [2].

The viruses causing gastroenteritis in humans include rotaviruses, caliciviruses (norovirus and sapovirus), astroviruses and enteric adenoviruses. Although some other viruses were identified in the gastroenteritis episodes in humans, their etiological roles have not been yet established [2].

Rotaviruses are the most common causes of viral gastroenteritis in children under 5 years of age. It has been estimated that they result in 440.000 cases of death a year in developing countries [4]. The progressive implementation of rotavirus vaccines in the field will hopefully change this picture. Typically, rotavirus gastroenteritis is more frequently observed in winter months under temperate climate conditions. Generally, its clinical course is considered to be more severe compared to the course of other viral gastroenteritis. It begins with a sudden onset of mild fever, vomiting and very loose stool; blood and mucus in stool are not seen at all. Vomiting lasts for 2-3 days and diarrhea is observed for 4-5 days on average [2,5].

Adenoviruses may cause epidemics, endemics and sporadic infections in all geographical regions of the world. The adenovirus type 40 and 41 cause gastroenteritis. The gastroenteritis caused by these types is mostly seen in the pediatric patients up to the 2 years old. They do not display seasonal distribution; and they can be seen throughout the entire year [6,7].

When compared to rotavirus infections, high fever and dehydration are less frequently observed and the infection duration is longer. It may be accompanied by vomiting and fever [7,8].

Definite diagnosis of viral gastroenteritis is important; although it does not change much the treatment strategy of the disease, it will decrease the unnecessary use of antibiotics. Especially in the outpatient clinics with more patient intensity, the rapid antigen tests have ensured rapid diagnosis with high sensitivity and specificity in several infectious diseases. The rapid antigen detection tests for rotavirus and adenovirus have begun to be used widely in clinical settings; and their use for the other viruses is also becoming widespread.

The trials about the viral agents of acute gastroenteritis are limited in our country. In our trial, we aimed to determine the frequency and to investigate the clinical manifestations of acute rotavirus and adenovirus gatroenteritis and to assess the diagnostic value of related clinical findings in patients admitted to Yeditepe University Hospital and the related health institutions.

## Methods

### Study Enrollment And Patients

All patients admitted with diarrhea and/or vomiting, diagnosed acute gastroenteritis and investigated for rotavirus or adenovirus antigens in stool, in Istanbul Yeditepe University Hospital and the related clinics between the years of 2007-2008, were enrolled into the study. Totally,1020 files were screened. The clinical data were collected by the study group through screening of the patient's electronic records, retrospectively. The patients who had insufficient data in the medical files were excluded from the study. 672 patients that had sufficient clinical data were included in the study. All of them were performed rotavirus antigen test and 596 of them were performed both rotavirus and adenovirus antigen test.

### Clinical Data

The patients' age, gender, chief complaints at referral, associated symptoms such as abdominal pain, antibiotic use, and clinical findings such as fever, vomiting, number of vomiting and diarrhea, dehydration, and the other pathological physical examination findings noted by the physicians were screened from the patient files, retrospectively. The chief complaints at referral were recorded as defined by their physicians and coded later on. Body temperature is measured with digital ear thermometer in our institution. Body temperature above 37, 5°C was accepted as "fever". Patients recorded as "dehydrated" by the physician or with the recorded physical findings of sunken eyes, dry tongue, reduced turgor-tonus and accompanying fatigue and activity impairment were considered as "clinically dehydrated".

### Laboratory

Stool samples were sent with Carry-Blair transport broth to the Microbiology Laboratory of Yeditepe University Hospital. CerTest Rota-Adeno Blister Test (CerTest, Biotec, Spain), a qualitative immunochromatographic assay was used to detect rotavirus and adenovirus antigens. In this test, the membrane in the test band was first coated with mouse monoclonal antibodies against viral antigens. During the test, the pre-colored conjugate that was previously dried on the test is reacted with the sample. Then, the mixture starts to move forward on the membrane by means of the capillary action. The colored particles are replaced as the sample moves on the test membrane. In case of a positive result, the specific antibodies on the membrane capture the colored particles.

### Statistics

The statistical analysis of the collected data was performed by using SPSS v.11.5 software. Descriptive statistics of data analysis have shown as percentages and ratios. In order to determine whether rotavirus positivity varied by various demographical and clinical characteristics of the patients in the study group, X^2 ^test was applied in the bivariate analyses, first. Then, multivariate analysis was performed using the Logistic Regression method. In the Logistic Regression analysis, presence or absence of rotavirus positivity was used as the dependent variable; and age, gender, polymorphonuclear leukocytes (PNL) in stool examination, month of referral, number of vomiting, number of stool, abdominal pain, fever and dehydration status were used as independent variables. The same statistical analysis was also performed to adenovirus positive group.

## Results

Rotavirus positivity was 18,7% (n = 126) among 672 patients included in the study. Adenovirus positivity was 8,9% (n = 53) in 596 patients whom adenovirus antigen test were also performed. The rotavirus-adenovirus coinfection rate was 4,4% (n = 26).

The Table 1 shows the distribution of the patients with gastroenteritis by the presence of rotavirus and various characteristics. As seen from the table, the presence or absence of rotavirus in the patients did not differ significantly by gender (p > 0.05). When considered with respect to the pediatric, the adult and geriatric age groups, rotavirus positivity was 21,5% in 0-14 pediatric age group, 13,7% in 15-64 adult age group and 18% in geriatric age group and there was a statistically significant difference between groups. Further statistical analysis indicated that, the difference was related to high rotavirus positivity in pediatric age group (p = 0.048).

**Table 1:** Distribution of rotavirus-positive and rotavirus-negative patients by various variables

Variables	Rotavirus-positive n (%)	Rotavirus-negative n (%)	Total n (%)
**Gender:**			

Male	65 (51.6)	289 (52.9)	354 (52.7)

Female	61 (48.4)	257 (47.1)	318 (47.3)

Total	126 (100.0)	546 (100.0)	672 (100.0)

	X^2 ^= 0.074	p = 0.785	

**Age group:**			

0-14	91 (72.8)	332 (61.1)	423 (63.3)

15-64	32 (25.6)	202 (37.2)	234 (35.0)

>64	2 (1.6)	9 (1.7)	11 (1.6)

Total	125 (100.0)	543 (100.0)	668 (100.0)

	X^2 ^= 6.087	p = 0.048*	

**Fever:**			

>37.5	29 (23.2)	92 (16.9)	121 (18.1)

37.5 and below	96 (76.8)	452 (83.1)	548 (81.9)

Total	125 (100.0)	544 (100.0)	669 (100.0)

	X^2 ^= 2.713	p = 0.100	

**PNL in Stool:**			

Yes	11 (9.0)	38 (7.2)	49 (7.5)

No	111 (91.0)	493 (92.8)	604 (92.5)

Total	122 (100.0)	531 (100.0)	653 (100.0)

	X^2 ^= 0.495	p = 0.482	

**Vomiting:**			

Yes	86 (68.8)	306 (56.3))	392 (58.6)

No	39 (31.2)	238 (43.8)	277 (41.4)

Total	125 (100.0)	544 (100.0)	669 (100.0)

	X^2 ^= 6.599	p = 0.010*	

**Abdominal pain:**			

Yes	14 (11.2)	78 (14.4)	92 (13.8)

No	111 (88.8)	462 (85.6)	573 (86.2)

Total	125 (100.0)	540 (100.0)	665 (100.0)

	X^2 ^= 0.896	p = 0.344	

**Dehydration:**			

Yes	31 (24.8)	41 (7.6)	72 (10.8)

No	94 (75.2)	501 (92.4)	595 (89.2)

Total	125 (100.0)	542 (100.0)	667 (100.0)

	X^2 ^= 31.335	p < 0.001*	

**Diarrhea-vomiting coexistence**			

Yes	53 (42,4)	162 (29,8)	215 (32,2)

No	72 (57,6)	381 (70,2)	453 (67,8)

Total	125 (100.0)	543 (100.0)	668 (100.0)

	X^2 ^= 7,351	P = 0.007*	

*: Statistically significant

High fever was determined in 23.2% of rotavirus cases and 16.9% of rotavirus-negative cases. However, no statistically significant difference was observed regarding high fever between rotavirus cases and the others (p > 0.05). Furthermore, no significant difference was observed among the groups in respect of presence of abdominal pain complaint (p > 0.05). On the other hand, a statistically significant difference was detected between the rotavirus cases and the rotavirus-negative cases regarding presence of vomiting and dehydration. The vomiting symptom (68.8%) observed in the rotavirus cases was statistically significantly higher than the other cases (56.3%) (p = 0.024). While diarrhea-vomiting coexistence was seen with a rate of 42,4% in rotavirus cases, the same rate was 29,8% in the other cases (p = 0.007). While clinical dehydration was present in 24.8% of rotavirus cases, it was 7.6% rotavirus-negative cases; and the difference between two groups was found to be statistically significant (p < 0.001).

The incidence rate of PNL existence in stool was determined as 9.0% for rotavirus cases and 7.2% for the others; accordingly, there is no statistically significant difference between rotavirus cases and the rotavirus-negative cases in respect of presence of PNL (p > 0.05).

The Table 2 presents the distribution of patients by their months of admission. As it can be seen from the table, there are significant differences among the patients regarding their months of referral (p < 0.001). Rotavirus cases were admitted in the months of December, January, February and March while the rotavirus-negative cases were admitted in the months of April, May and July.

**Table 2:** Distribution of rotavirus-positive and rotavirus-negative patients by their months of admission

Month of admission	Rotavirus-positive n (%)	Rotavirus-negative n (%)	Total n (%)
1	16 (12.7)	42 (7.7)	58 (8.6)

2	22 (17.5)	42 (7.7)	64 (9.5)

3	14 (11.1)	43 (7.9)	57 (8.5)

4	8 (6.3)	76 (13.9)	84 (12.5)

5	9 (7.1)	52 (9.5)	61 (9.1)

6	7 (5.6)	41 (7.5)	48 (7.1)

7	10 (7.9)	56 (10.3)	66 (9.8)

8	7 (5.6)	40 (7.3)	47 (7.0)

9	3 (2.4)	41 (7.5)	44 (6.5)

10	8 (6.3)	34 (6.2)	42 (6.3)

11	4 (3.2)	40 (7.3)	44 (6.5)

12	18 (14.3)	39 (7.1)	57 (8.5)

Total	126 (100.0)	546 (100.0)	672 (100.0)

	X^2 ^= 34.359	p < 0.001*	

*: Statistically significant

The Table 3 shows the multivariate analysis results between the rotavirus-positive cases and the others. According to the logistic regression analysis results, clinical dehydration was the most important variable regarding presence or absence of rotaviruses in the patients. Accordingly, presence of clinical dehydration (p = 0.010) is the most important determinant for the presence of rotavirus in the cases.

**Table 3:** Relationship between various variables and the rotavirus-positive and rotavirus-negative patients according to the logistic regression analysis results

	B	**S.E**.	**Sig**.	Exp(B)
Age	-,237	,134	,075	,789

PNL	,726	,543	,181	2.067

Gender	-,072	,343	,833	,930

Month of admission	-,005	,050	,918	,995

Number of vomiting	-,021	,060	,731	,980

Number of stools	,034	,046	,455	1,035

Abdominal pain	-,380	,540	,481	,684

Dehydration	1,213	,471	,010*	3,364

Fever	,012	,013	,349	1,012

Constant	-1,556	,734	,034	,211

*: Statistically significant

In the bivariate analyses, there was a statistically significant difference regarding vomiting and dehydration observed in rotavirus-positive cases of diarrhea compared to the rotavirus-negative cases. However, only dehydration was determined as an important variable in the multi-regression analyses.

Since the number of patients with adenovirus-positive gastroenteritis was low in our sample and half of these patients were also rotavirus-positive, no statistical significance was determined for any variable.

## Discussion

Acute gastroenteritis is considered one of the most common causes of admission to health centers. Viral pathogens are frequently found in the etiology. Viral gastroenteritis is usually diagnosed by ruling out the other agents according to the clinical findings or the results of the available tests. The antigen tests used in diagnosis of viral gastroenteritis are easy to use and offer high sensitivity and specificity. Therefore, they provide best alternatives to be used in diagnosis, today [2].

Rotavirus antigen test positivity was 18,7% in the entire sample. The studies carried out in our country usually involved the pediatric age group; the trials performed in adult groups are limited. The rotavirus positivity varied in the range of 20%-37% although the age groups and the trial methods were not homogenous in these studies [9-11]. Furthermore, the studies in the literature have also focused on the pediatric age group due to the high incidence and morbidity and mortality risk in this age group. In the trials carried out in different countries, rotavirus positivity varies between 20%-50% in different pediatric age groups [12-18]. In the trial carried out in Southern Korea by Huh et al [19] with 10.028 stool samples collected from all age groups, the viral agent rate was determined as 29% with rotavirus frequency of 19.3% and adenovirus frequency of 0.007% among all viral agents. This rate is very close to the rotavirus positivity that we have determined in our study. Regarding these data, although rotavirus gastroenteritis is more common in the children under 5 years of age, it can be seen with a definite frequency in every age group.

In the patients for whom adenovirus rapid antigen detection test was performed, the rate of positive cases was 8,9%. In the literature, it is stated that adenovirus accounts for 5-20% of diarrheas observed in the pediatric age group [2]. In the studies carried out in the pediatric age group in our country, the rates between 4,7% - 16,2% were reported [9,20,21].

Co-existence of more than one agent is not rare in gastroenteritis cases. Although bacteria-virus coinfection may also be observed, it is generally seen in the form of virus-virus coexistence. Rotavirus-adenovirus coexistence was detected in 4,4% of our patients whom both rotavirus and adenovirus antigen tests were performed. In the trial carried out by Biçer et al., this rate was found as 4,4%; and it is very close to the rate that we have determined.

As it was expected, rotavirus cases were more common between the months of December - March. While frequency of rotavirus cases may vary by geographical location, most cases are observed in the winter months under temperate climate conditions. In the studies carried out in our country, it was reported that these cases were more frequently seen in the months of September and November as reported by Bulut et al. [22], in the autumn months as reported by Şimşek et al. [23], in the months of December, January and February as reported by Biçer et al. [9], in the months of December - April as reported by Karadağ et al. [10], and in the months of January, February and March as reported by Nazik et al. [11]. In the Asian rotavirus surveillance study, it was reported that it was observed with a higher frequency between the months of December - March in the northern regions, but such a seasonal increase was not observed in the countries with tropical climate such as Vietnam, Thailand, Malaysia, etc [12]. In the study carried out by Jones et al. with 224160 children in Canada, it was reported that it was observed with a higher incidence in the months of April - May [24]. Geographical location seems to be important regarding seasonal variability of rotavirus-related gastroenteritis. There are no adequate data related with seasonal variability in different regions of our country. Based on available data, it can be stated that it is more frequently observed in the winter months.

Vomiting, dehydration and vomiting-diarrhea coexistence were observed more frequently in rotavirus-positive gastroenteritis cases compared to the rotavirus-negative cases. In the study by Coffin et al., they showed that rotavirus gastroenteritis was more related with fever, vomiting and diarrhea-vomiting coexistence [18]. Presence of fever is one of the most important clinical findings affecting the clinician's decision in the approach to the infectious diseases. In the clinical studies, it has been reported that fever is observed more frequently in rotavirus gastroenteritis compared to the other viral agents [14,18,25-27]. We did not find any difference regarding fever between the rotavirus cases and the rotavirus-negative cases in our study. In the study carried out by Şimşek et al., no difference was determined between the rotavirus-positive and rotavirus-negative patients according to the rotavirus diarrhea scoring system defined by Ruuska and Vesikari [23]. It is known that rotavirus gastroenteritis has a more severe clinical course and requires more hospitalization and intravenous fluid treatment [3]. The course of rotavirus-related gastroenteritis with more vomiting and dehydration and more frequent vomiting-diarrhea coexistence may be alarming for the clinician. However, it is not possible to differentiate rotavirus from the other agents merely on the basis of clinical findings [25,26].

In the stool direct microscopy of gastroenteritis cases, PNL existence classically suggest inflammatory bacterial gastroenteritis [28,29]. PNL can also be seen in the direct microscopy of viral gastroenteritis cases. In the literature, PNL existence in stool was reported up to 30% in rotavirus gastroenteritis cases [5]. This rate was 9% in our cases. Probably, there were also some other viral agents except that adenovirus in the rotavirus-negative group. It is not possible to make a viral-bacterial comparison on basis of these data. Regarding treatment approach, it is important to know that, PNL existence in stool is not a gold standard for bacterial - viral agent differentiation; it may also be seen in viral cases.

Our study has some limitations. The data do not cover all gastroenteritis cases attending to our hospital; and the clinicians have decided on rapid antigen test and/or culture according to their clinical findings and opinion. The data were collected from the patient records stored in electronic medium and the missing data in some files about some parameters such as number of vomiting, etc. may have affected our results.

## Conclusion

Rotavirus can be seen in all age groups, but more frequently in childhood. Although there is no clinical gold standard to distinguish the rotavirus cases from the other gastroenteritis agents, the findings of dehydration and vomiting-diarrhea coexistence, considering months of referral may lead clinician to perform rapid antigen tests and affect approach to the treatment. Prospective studies with representative samples are needed to determine the rotavirus and adenovirus incidence and to develop safe and reliable protective policies in our country.

## Competing interests

The authors declare that they have no competing interests.

## Authors' contributions

HA carried out the design and coordination of the study and data entrance and analysis. Also participated in the sequence alignment and drafted the manuscript; GI carried out data entrance and analysis and in the sequence alignment and drafted the manuscript; YG carried out the rapid antigen tests and other laboratory evaluation and data entrance; SS participated in data entrance and analysis; TSG carried out the rapid antigen tests and other laboratory evaluation and participated in data entrance; GY participated in its design and coordination and helped to draft the manuscript. She was also scientific consultant of the study; OH performed the statistical analysis. FG conceived of the study, and participated in its design and coordination and helped to draft the manuscript; AV participated in its design and coordination and helped to draft the manuscript. She was also scientific consultant of the study. All authors read and approved the final manuscript.
